# Electrical resistivity tomography data across the Hockai Fault Zone (Ardenne, Belgium)

**DOI:** 10.1016/j.dib.2016.12.028

**Published:** 2016-12-21

**Authors:** Thomas Lecocq, Thierry Camelbeeck

**Affiliations:** Royal Observatory of Belgium, Belgium

**Keywords:** ERT, Fault zone, Resistivity, 3D

## Abstract

In this work, we present the result of a large-scale geophysical survey that had the objective of identifying the subsurface characteristics and the NE–SW extension of the Hockai Fault Zone: a major NNW–SSE oriented crustal-rooted fault zone crossing the Stavelot-Venn Massif (Eastern Belgium). 31 two-dimensional electrical resistivity tomography (ERT) profiles are presented, resulting in 10,679 m of 2D sections. All profiles were acquired between 2008 and 2010 using a single channel ABEM Terrameter SAS1000 instrument connected to a 64 electrodes setup of maximum 315 m extent which was often extended using the roll-along technique. Major findings based on the data presented here are reported in the manuscript "A geophysical cross-section of the Hockai Fault Zone (Eastern Belgium)" (Lecocq and Camelbeeck, Submitted for publication) [1].

**Specifications Table**

**Table:** 

Subject area	*Geophysics*
More specific subject area	*Subsurface geophysical imaging*
Type of data	*ABEM AMP, Res2Dinv DAT, text file, PNG figure, 3D vtk/vtu and KMZ*
How data was acquired	*ABEM Terrameter SAS1000 for the Electrical Resistivity data*
	*Leica Total Station TC1800 for the topographical data*
Data format	*Raw and Processed*
Data source location	*Site: Hockai Fault Zone*
	*Region: Ardenne*
	*Country: Belgium*
	*Coordinates: 50.48 N, 5.97 E*
Data accessibility	The data is with this article.

**Value of the data**

• The data presents the largest ERT geophysical survey across the Hockai Fault Zone.
• The profiles allow identifying subsurface features, such as stratification in geological units, faults and wet zones.
• We publish these data so they can be consulted in future research in the study area and/or for teaching purposes.
• Raw data can be reprocessed using different 2D or even 3D inversion schemes.

## 1 Data, experimental design, materials and methods

### 1.1 Data acquisition

**Fig. 1:**
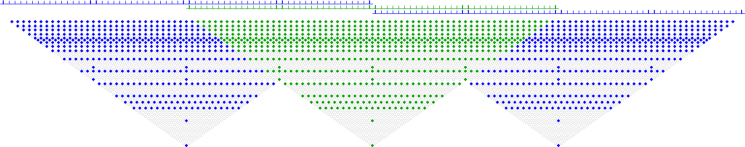
SCH-ORB3 acquisition protocol used for all ERT acquired on the HFZ. This figure shows a standard profile with 901 points and two "roll-along" extensions.

**Fig. 2:**
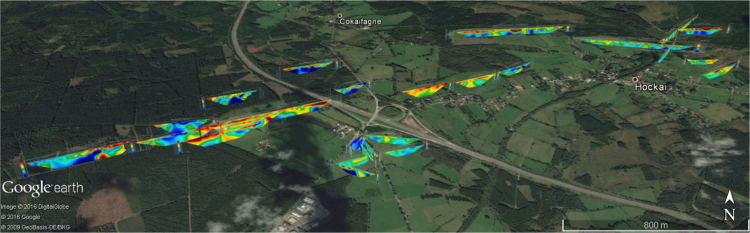
3D view of the electrical resistivity tomographies across the Hockai Fault Zone.

**Electrical resistivity tomography** (ERT) is a relatively fast method yielding a vertical profile of resistivity variation in the subsurface. The principle is to deploy a number of electrodes at regular spacing along a straight line, all connected to an electric cable that on its turn is connected to a computer (the Terrameter). Afterwards current is injected, potential is measured from which apparent resistivity is deduced. Apparent resistivities are subsequently inversed to obtain a 2D resistivity profile. The full theory and practice of the method are described by Loke [2]. The ideal conditions for the method to be fully valid are that the medium investigated is mostly 2D, i.e. that lateral variations relative to the profile position are negligible. A perfect case would be a layered cake (layers over half space), crossing any structure perpendicularly. In our data, this kind of case is sometimes met but mostly not (Fig. 1, Fig. 2).

All profiles have been acquired using a hybrid Wenner-Schlumberger protocol of 901 measurements for each 64 electrode profile. The hybrid protocol was chosen to be sensitive to both vertical and horizontal resistivity changes [2]. The equipment used was a single channel ABEM Terrameter SAS1000. When possible, extensions (roll-along technique) were performed in order to increase the profile in length to 1.5, 2 or 2.5 times its standard extent. The maximal extent of a 64 electrode profile is 315 m, i.e. a maximum electrode spacing of 5 m. Extending, or rolling-along, allows prolongation of the profiles *n* times to obtain longer profiles of total length of 315 *(1+*n*/2) (Table 1).

#### 1.1.1 Georeferencing

The relative 3D location of the electrodes was measured with a Leica TP1800 total station. The location of the total station was measured using a handheld GPS unit and the final location of the profiles was obtained by translating the relative coordinates to WGS84 latitude/longitude/altitude values, carefully checking their location and orientation on georeferenced maps and photos. The final horizontal error of the location of each profile ranges between 1 m and 5 m, while the relative location of electrodes within each profile is lower than 5 cm.

#### 1.1.2 Location and timing

The data was acquired during three campaigns: October 2008, August 2009 and August 2010, i.e. during Thomas Lecocq׳s PhD [1], [3]. The field is located in the vicinity of Hockai and Francorchamps, in eastern Belgium on the Vecquée Crest, the higher crest of the Venn area. The subsurface geology is poorly known due to the lack of boreholes/outcrop availability but supposed to be composed of a mix of steeply dipping Cambrian quartzites and phyllads with layer thicknesses ranging from 1 to 10 m. The stratification is generally oriented N040 to N070, with a SE-dip of 45–80°. Most of the ERT profiles intersects this direction with a relatively small angle. This orientation was chosen to cross-cut the Hockai Fault Zone at a maximum perpendicular angle, i.e. along a northeast-southwest cross-section, which results in an apparent dip of the stratification.

### 1.2 Processing

ERT data has been processed using RES2DINV. Data points with relative error larger than 2.5% are discarded. The inversion is done using three different schemes, namely a "normal" scheme (L2 norm, standard least-square constraint), a "robust" scheme (L1 norm) and a "combined" scheme (Combined Marquardt and Occam inversion method). The three outputs are presented with this article. The standard least-square constraint (L2) attempts to minimize the square of the differences between the observed and calculated apparent resistivity values, the robust constraint (L1) is less sensitive to very noisy data points but might give higher apparent resistivity RMS error. In practice: if the subsurface resistivity changes in a smooth manner, one should use the standard least-squares constraint. If there are sharp boundaries, one should choose the robust model inversion constraint. The Combined Marquardt and Occam inversion method combines the Marquardt (or damped least square method) with the smoothness-constrained method. It seems to give better results in resolving compact structures where the width and thickness are slightly smaller than depth, such as a cave or an ore-body [4] and RES2DINV GUIs.

Usually, the L2-norm yields smoothly varying resistivity "blobs" while the L1 yields blocky profiles with horizontal and vertical gradients. The combined scheme yields intermediate results. For each profile, the inversion was stopped when the improvement in the RMS misfit was not significant. Most profiles reach a satisfying RMS misfit after 3–6 iterations, while few needed to reach iteration 9.

Resistivity values are mostly comprised between 100 and 2000 Ω m. Those values are present at random locations along the profiles and at depth.

## 2 Processed data in supplementary files

All profiles are provided in RES2DINV default output format (INV), in a PNG format with or without a fixed common color scale, in XYZ-R ascii files and in VTK/VTU 3D sections. The common color scale profiles for the "normal" inversion (L2 norm) are also presented as 3D models in KMZ format using the technique of Van Noten [5].

## Figures and Tables

**Table 1:** Wenner-Schlumberger (WSC) parameters "*n*" and "*a*" used in the SCH-ORB3 acquisition protocol.

WSC	*n*=1.0	*a*=[1,3–5,7,9,11,13]
WSC	*n*=2.0	*a*=[1]
WSC	*n*=3.0	*a*=[1,6]
WSC	*n*=4.0	*a*=[1,3,5]
WSC	*n*=5.0	*a*=[1,3]
WSC	*n*=6.0	*a*=[1,3]
WSC	*n*=7.0	*a*=[1]
WSC	*n*=8.0	*a*=[1]
WSC	*n*=10.0	*a*=[1]
